# Nuances in the global impact of COVID-19 on tuberculosis control efforts: An updated review

**DOI:** 10.1097/MD.0000000000042195

**Published:** 2025-04-18

**Authors:** Kiavash Semnani, Shirin Esmaeili

**Affiliations:** a Tehran University of Medical Sciences School of Medicine, Tehran, Iran; b Tehran University of Medical Sciences School of Medicine, Tehran, Iran.

**Keywords:** COVID-19, incidence, mortality, pandemic, tuberculosis

## Abstract

The COVID-19 pandemic has affected public health systems in an unprecedented manner. There has been an abundance of discussion regarding the possible effects of the pandemic in disruption of health services aiming at tuberculosis (TB) infection control – including hindered screening efforts and delays in diagnosis and treatment. The pandemic has also been proposed to affect TB transmission via lifestyle modifications. Moreover, some research has suggested a more direct link between COVID-19 infection and increased TB morbidity and mortality. The authors conducted a narrative review of the relevant literature. Searches were performed in the MEDLINE, Scopus, and Web of Science databases. Reports of impaired TB case-notification were ubiquitous during the early stages of the pandemic. Subsequently, divergent patterns emerged: recovery and decreased TB incidence in countries with stringent public health measures, low local transmission of TB, and resilient health systems; or devastating results from TB underdiagnosis and delayed treatment in countries with high TB burden, limited COVID-19 control measures, and public health funding. Few studies quantified the effects of TB and COVID-19 co-infection – and the possible role of COVID-19 infection in reactivation of latent tuberculosis infection (LTBI) remains ambiguous. Despite the lapse of the COVID-19 pandemic, its effects on TB control efforts perseverate. Particularly, great care is warranted for recovery of impacted healthcare systems in low-income countries.

## 1. Introduction

### 1.1. Tuberculosis: the “white death”

Tuberculosis (TB) has continued to plague the human species throughout centuries.^[1,2]^ Even today, TB is the leading cause of death related to infectious disease – a title it had momentarily relinquished to COVID-19.^[3]^ Airborne transmission along with the infection’s tendency to go unnoticed for months or years (latent infection) have led to its integration with human civilization.^[4]^ Currently, latent tuberculosis infection (LTBI) is estimated to affect more than a third of the world’s population, with 5% to 15% of these cases expected to progress to disease (reactivate).^[5]^

TB spread has historically benefited from disasters – wars and epidemics.^[1]^ Particularly, surges in viral diseases such as the 1918 flu epidemic, or more recently, SARS and MERS outbreaks have been linked to increased TB mortality.^[6]^ One explanation for this may be the toll an epidemic takes on those already at-risk for TB – via promotion of poverty and, in turn, increased malnutrition and overcrowding.^[7]^ Similarly, disruptions in public health systems inadequately prepared for epidemics, increase the risk of TB spread by missed opportunities for diagnosis and treatment.^[8]^ Furthermore, in some cases, the emerging disease may directly impact the host’s immune system – and possibly mechanisms of adaptive immunity specific to TB control (e.g. CD4 response) – (“viral immunosuppression”), enhancing chances of TB reactivation, or more catastrophic consequences of active tuberculosis; this is perhaps best demonstrated by the effects of HIV infection (and later epidemic) on TB.^[1,9,10]^

### 1.2. Proposed impact of the COVID-19 pandemic on TB control efforts

**Figure 1.**
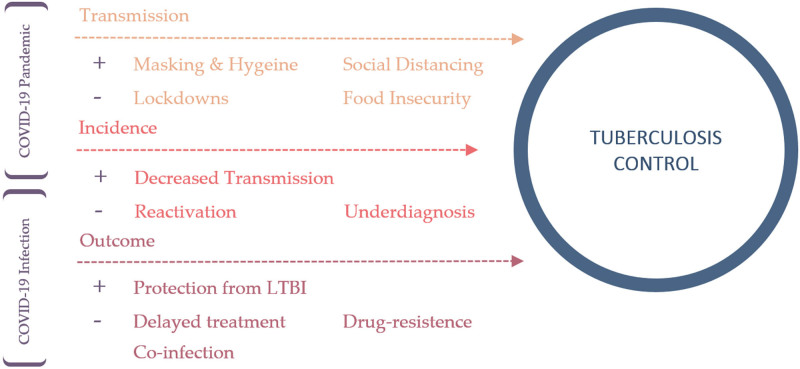
Proposed impacts of COVID-19 on TB control. + = exacerbation; − = attenuation; LTBI = latent tuberculosis infection.

The World Health Organization (WHO) declared the COVID-19 pandemic on March 11, 2020.^[7]^ Soon thereafter, health systems began directing their attention and funding towards COVID-19 control – diverting resources from programs focusing on TB, HIV, malaria, and neglected tropical diseases.^[11]^ This, along with the public’s newly formed fear of care-seeking, led to disruptions in a majority of TB programs – as manifested by a 25% to 30% early reduction in TB notifications.^[12]^ Figure 1 depicts possible impacts of COVID-19 on TB control.

#### 1.2.1. Case identification and treatment

In the early months of the pandemic, reports indicated a concerning development of decreased TB diagnoses in contrast to increased positivity rate for tests performed, severity of cases, loss of patients to follow-up, and mortality.^[13–16]^ This suggested that unlike several other respiratory diseases (e.g. influenza), the decrement in TB diagnoses may be due to underdiagnosis rather than a true reduction in TB incidence.^[13]^ Diminished access to TB care during lockdowns, and lack of attention to TB diagnosis in the presence of overlapping symptoms with COVID-19, along with severe degradation of community-based TB care (outreach initiatives, mobile clinics, etc.) were designated the chief culprits.^[6,12]^ During the same period the WHO recommended a shift toward home-based treatments to decrease the need for less-available in-person visits – at the risk of questionable adherence with treatment side effects and subsequent promotion of drug-resistant TB (DR-TB).^[17]^

By the end of the first surge of COVID-19, what remained certain was the decrease in TB case notifications.^[18]^ Reports from the WHO European region showed a 35.5% drop in case diagnosis and treatment by the second quarter of 2020.^[19]^ Similarly, there were 25% less cases notified from India, the Philippines, and Indonesia (three countries with a high-burden of TB) during the first half of 2020, along with disruptions in directly observed treatment (DOT) and drug supplies – enhancing the possibility for spread of DR-TB.^[7]^ A December 2020 report from 19 countries, also, indicated decreased case identification in almost all 43 TB centers studied.^[20]^

#### 1.2.2. Public health measures and TB transmission

Several studies hypothesized the drop in case notifications may, in fact, be a true reflection of decreased TB incidence and transmission following the implementation of public health measures such as lockdowns, social distancing, and broader use of masks and sanitizers during the early phases of the COVID-19 pandemic.^[18]^ However, even if such measures did decrease TB transmissions, the decrease in TB incidence would be reasonably delayed in light of TB’s prolonged latency period.^[1]^ This fact supports the fall in TB notifications being attributable to underreporting.^[13]^ It was also proposed that lockdowns may lead to increased household transmission of TB due to prolonged close-contact. This mode of that constitutes a small proportion of transmissions, but disproportionately affects children and people living in overcrowded settings (e.g. refugee camps, urban slums, and prisons)–due to higher prevalence of intimate and prolonged exposures.^[1,4]^ To this affect, an early pandemic study from Spain found increased rates of LTBI and acute TB in children from affected households.^[15]^ Furthermore, there is a paucity of evidence suggesting that the use of surgical masks, similar to those used during the pandemic, reliably prevents TB transmission beyond what had been observed in small scale studies and animal models.^[21]^

#### 1.2.3. TB/COVID-19 co-infection

Initial reports indicated worse outcomes for TB/COVID-19 co-infection when com-pared to each infection.^[22]^ A scoping review by Flores-Lovon et al found host immune responses to be enhanced in patients manifesting COVID-19 and LTBI, with higher COVID-19 specific neutralizing antibodies, absolute lymphocyte counts, and IFN-γ levels. Meanwhile, patients with acute TB and COVID-19 manifested lower CD4 polyfunctional capacity, and IFN-γ response to a SARS-Cov-2 spike protein antigen.^[23]^ However, poorer outcomes of co-infection may have also been attributable to higher prevalence of risk factors associated with COVID-19 mortality (low socioeconomic status, diabetes, renal disease etc.) among TB patients.^[24]^

#### 1.2.4. COVID-19 and reactivation of TB

Multiple cases of TB reactivation in the presence of prior COVID-19 history were presented during the early pandemic era.^[25–27]^ Impaired TB-specific CD4 response, vessel wall ingrowth of granuloma, and increased generalized pulmonary inflammatory response to TB were also observed in patients with COVID-19.^[22,28]^ Similarly, a mouse model for TB showed promotion of intracellular TB replication via activation of CD271 “altruistic” stem-cells in the presence of murine coronavirus infection.^[29]^ Furthermore, the use of immunosuppressive drugs such as corticosteroids and Tocilizumab for treat-ment of severe COVID-19 was suggested to entail a risk of opportunistic infections and TB reactivation.^[30,31]^ However, the extent to which these concerning propositions may translate to increased real-world incidence of TB was scarcely explored.

### 1.3. Review objectives

The 2020 WHO global tuberculosis report estimated disruptions caused by the pan-demic to cause an excess 13% mortality in the coming years.^[7]^ However, a 2021 Global Burden of Disease (GBD) study reported a more conservative estimate of the pandemic’s negative impacts – citing the divergence of the WHO assumption of no true decrease in incidence of tuberculosis with developing data.^[32]^ We aimed to seek TB rates reported in the literature, and contrast them with contextual indicators of true changes in incidence. The temporal relationship between local public health measures/disruptions as well as socioeconomic changes with resulting TB reports was explored. Similarly, we sought evidence on outcomes for TB/COVID-19 co-infection and reports of post-COVID-19 tuberculosis, and weighed host risk factors against immunological interpretations of the results.

The authors aimed to revisit aforementioned hypotheses regarding impacts of the COVID-19 pandemic and infection on TB control efforts. And to explore the extent of evidence available for each proposition in a post-pandemic era. We hope this review will provide a more accurate perspective on the nuances of TB diagnosis trends as impacted by the pandemic, and adjust assumptions in modeling changes in TB burden yet to be observed. Alternatively, we intend to highlight the role of underreported factors influencing such interpretation.

## 2. Materials and methods

The current is a narrative review of literature on the effects the COVID-19 pandemic and infection have had on TB control efforts. The authors opted to use narrative in lieu of a systematic approach to highlight the range of possible impacts the COVID-19 pandemic has had on TB control efforts. The SANRA scale for quality assessment of narrative reviews was used to guide the review’s structure. However, no assessment was conducted to evaluate included studies for risk of bias.

### 2.1. Search strategy

A search of the MEDLINE, Scopus, and Web of Science (WoS) electronic databases was conducted on November 06, 2024. Equivalent MeSH terms were used to maximize search yield. Search queries used are presented in Table 1. Search queries were adjusted to result all studies published in English after 2019. Identified records were managed in EndNote X9. Selected studies were categorized for reporting based on reported effects of COVID-19 on TB control.

**Table 1:** Search queries used for each database.

Database (interface)	Search query
MEDLINE (PubMed)	(COVID[Title/Abstract] OR COVID-19[Title/Abstract] OR COVID19[Title/Abstract] OR 2019-nCoV[Title/Abstract] OR SARS-CoV-2[Title/Abstract] OR pandemic[Title/Abstract] OR coronavirus[Title/Abstract]) AND (TB[Title/Abstract] OR Tuberculosis[Title/Abstract] OR Mycobacterium[Title/Abstract] OR mycobacterial[Title/Abstract] OR Koch[Title/Abstract])
Scopus	TITLE-ABS ((COVID OR COVID-19 OR COVID19 OR coronavirus OR SARS-CoV-2 OR 2019-nCoV OR pandemic) AND (TB OR tuberculosis OR mycobacterium OR mycobacterial OR Koch))
WoS	AB=((TB or Tuberculosis or mycobacterium or mycobacterial or Koch) and (COVID or COVID-19 or COVID19 or coronavirus or SARS-CoV-2 or 2019-nCov or pandemic)) OR TI=((TB or Tuberculosis or mycobacterium or mycobacterial or Koch) and (COVID or COVID-19 or COVID19 or coronavirus or SARS-CoV-2 or 2019-nCov or pandemic))

WoS = Web of Science.

### 2.2. Inclusion and exclusion criteria

Subsequent to the search, each article’s title and abstract were screened by two independent reviewers for relevance. Studies without relevant data or opinions on TB incidence, transmission, reactivation, and outcomes were excluded. Research protocols and qualitative studies were also excluded. Narrative reviews, opinions (editorials, perspectives, etc.), and modeling studies were included in full-text review with the aim to extract accompanying or underlying data. Early case-reports and case-series with higher numbers of co-infection or post-COVID-19 TB cases were included considering a paucity of original research on the topics. Studies focusing solely on tangential relationships such as TB-HIV-COVID-19, TB-Diabetes-COVID-19, and BCG-COVID-19 were excluded, as were studies reporting interventions aimed at improving TB control. Finally information was extracted from relevant full-texts and categorized according to the impact focused on and whether the study contained mere hypotheses or carried supporting data. Studies with few or no supporting data, as well as early pandemic studies were used to present each topic (in Introduction), while results from large-scale original studies were selected to be reported in Discussion.

### 2.3. Ethical considerations

The presented research is based on previously approved and published literature. No sensitive patient data was retrieved or reported. Permission to use information from published works was obtained according to the relevant licensing.

## 3. Results

**Figure 2.**
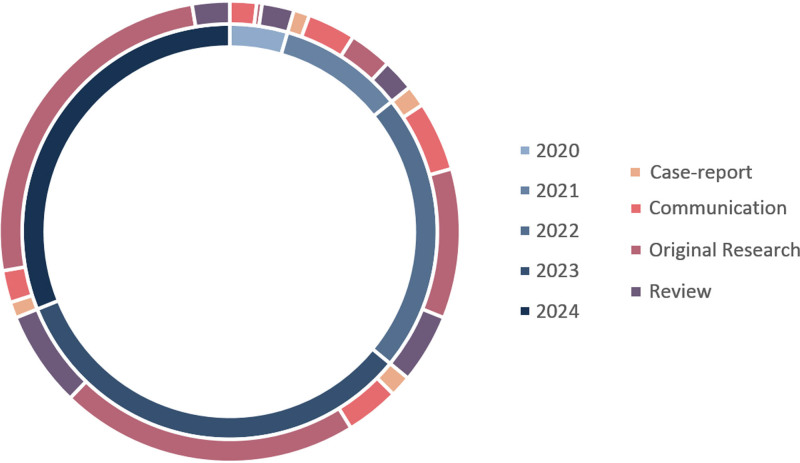
Proportions of each article type reviewed by year of publication.

The search in MEDLINE yielded a total of 2991 title/abstracts, and the searches in Scopus and WoS resulted in 3437, and 2914 results, respectively. After exclusion of duplicate results and title and abstract screening a total of 267 full-text articles were reviewed. Figure 2 depicts the proportions for each article type reviewed by publication year. Altogether, 160 original articles, 50 narrative or systematic reviews, 43 communications, and 14 case-report/case-series were reviewed. Included studies encompassed a great range of geographic expansion – providing consideration of COVID-19 effects on TB both in low and high-burden countries.

## 4. Discussion

### 4.1. TB case notifications and treatment

#### 4.1.1. Trends in TB case notifications

Reports from early waves of the pandemic indicate an almost universal decrease in TB testing and case identification.^[20]^ Testing was found to be impaired throughout 2020 in France, Italy, Spain, and the United Kingdom.^[33]^ Over 90% of districts in Indonesia, a country with one of the highest TB burdens, reported 50% to 75% reduction in their budget with a subsequent 60% drop in case notifications.^[34]^ Similarly, India reported a 78% in case notification during the first wave of the pandemic while Japan, South Korea, and Taiwan experienced less disruptions.^[35]^ Winglee et al., compared the US National TB Surveillance System data with pharmacy data, and found no underreporting of diagnoses made – showing the > 20% drop in registered cases by November 2020 was either a result of underdiagnosis, or a true decrease in incidence.^[36]^

In the same time period several studies reported an increase in positive rate of TB tests with less tests performed. This may be a consequence of only more severe or symptomatic cases (with a higher yield) being tested.^[37–40]^ Stantliff et al, found the 60% de-crease in testing in 2020 (Ohio, USA) to be more severe among asymptomatic persons screened due to migration.^[41]^ A study from Almaty, Kazakhstan, also found a shift in diagnosis from asymptomatic screening to symptomatic presentations, along with the 23.2% and 31.2% drops in diagnosis for 2020 and 2021, respectively.^[42]^ Meanwhile, a study from Nigeria found a less severe drop (2.4%) in cases identified from 2020 to 2022 due expanded screening efforts.^[43]^ The overlap in COVID-19 and TB symptoms may have also contributed to underdiagnosis of TB via misdiagnoses.^[44]^

Further supporting the possibility of underdiagnosis during the early stages of the pandemic, 73% of Indian TB centers indicated workload shift towards COVID-19 control efforts – especially in areas of community outreach and molecular testing.^[45]^ A survey of 40 international TB labs found 22.5% of them reporting negative impacts on standard operations in 2020 and 2021.^[46]^

The following trends in case notifications were divergent. Reports from India, South Africa, and Zambia showed recurrent drops in TB diagnosis with surges in COVID-19, more so, during the delta variant’s peak circulation.^[8,47–48]^ Surveillance data from China, however, showed a maximal drop in notification in 2020 followed by sustained recovery of the number of cases – never above 2019 levels.^[49]^ Similarly, reports from Italy and Taiwan presented a sustained decrease in cases reported throughout the pandemic – suggesting a lasting effect of implemented public health measures.^[50,51]^ Results from two retrospective studies based in India, also, showed a post-pandemic decrease in TB cases without increased mortality from underdiagnosis.^[52,53]^

Several countries reported an increase in TB cases reported after the pandemic. Re-ports from Brazil showed a recovery in case identification in 2021,^[54]^ followed by an in-crease in cases in 2022.^[55]^ Similarly, data from East Java Indonesia and morocco showed, respectively, a 19.9% and 53.1% drop in diagnosed cases during the pandemic, and a subsequent 31.9% and 65.3% increase post-pandemic.^[56,57]^ Global notifications report-ed to the WHO followed a similar pattern with 7.5 million cases in 2022 to 400 thousand more than that of 2019.^[58]^

Ledesma et al.’s model of the pandemic’s impact on TB diagnosis showed the African region outperforming the model in 2021 in ratio of observed to expected cases (O/E) (O/E = 0.97 in 2020 and 1.02 in 2021), while global results showed a broader, yet diminishing impact (O/E = 0.79 in 2020 and 0.83 in 2021). The high-burden region of Southeast Asia, however, showed the worst trend (O/E = 0.74 in 2020 and 0.71 in 2021).^[59]^ It was suggested that stringent public health measures during the pandemic may have slashed TB transmission by 50%, but such measure were only applied in limited scope.^[60]^ In a model utilizing data from the National Health Commission of the People’s Republic of China, TB incidence for the 2020 to 2023 timeframe consistently outperformed predictions while other respiratory diseases showed lower incidence than expected – suggesting low-er, and perhaps, delayed influence of public health measures on TB.^[61]^ Oh et al, analyzed different dynamics in case notifications in the Western Pacific region. Despite a global 21% and 23% decrease in case notification in 2020 and 2021, respectively, an 18% in-crease in cases was observed for 2022. Younger individuals compromised the bulk new-ly-diagnosed. Furthermore, countries with a lower Human Development Index (HDI) exceeded predicted and pre-pandemic TB rates while countries with more stringent public health measures (China, Hong Kong, and Japan), or those with low TB burden implementing travel restrictions (Australia, New Zealand), showed steady decline in TB diagnosis.^[62]^

#### 4.1.2. Drug resistant tuberculosis

Drug-resistance is the ability of bacteria to resist medications that have earlier demonstrated efficacy in their treatment.^[63]^ DNA mutations can cause resistance to evolve naturally over time, while horizontal gene transfer can allow bacteria to acquire resistance from other resistant bacteria. Resistance is accelerated by elements including the overuse and misuse (such as partial treatment) of antibiotics in agriculture, human health, and animal husbandry. Resistant Mycobacterium tuberculosis provide a challenge in both treatment and prevention of transmission for TB.^[64]^

Few reports have focused on the prevalence of DR-TB during the pandemic. Most studies showed a decrease in absolute number of DR-TB cases identified that was proportional to changes in overall TB notifications.^[65–67]^ Meanwhile, A 5-year analysis performed in the Henan province of China showed decrease in DR-TB from 2017 to 2020 (19.1–15.7%) with a minor resurgence in 2021 (17.5%).^[68]^ A study from Nigeria found a similar late-pandemic resurgence in DR-TB.^[43]^ However, it is important to note changes in the prevalence of DR-TB may not reflect solely the pandemic’s impact on TB services, as several breakthroughs in management of DR-TB (e.g. increased case referral, decreased quinolone abuse, and more common use of Linezolid) have occurred during the same period.^[68]^

#### 4.1.3. TB treatment

Treatment coverage for diagnosed cases during the pandemic seems to have suffered less than case identification^[62]^; global results show only 11% and 7% drops in 2020 and 2021, followed by a 5% increase in coverage for 2023.^[3]^ A US-based study showed no impact on the proportion of patients treated,^[41]^ and reports from Peru indicated a mere 2.5% dropout during 2020.^[69]^ In contrast, more severe impacts were observed in Indonesia (10–20% decrease in treatment),^[34,70]^ and Mexico (46.7%).^[71]^

Orfào et al, reported a 50% drop in DOT in Paraná, Brazil during the early pandemic period.^[72]^ Lockdowns were also found to be associated with increased DOT non-adherence (relative risk (RR) = 1.42, 95% confidence interval (CI): 1.04–1.96).^[73]^ One study showed poorer outcomes of treatment with delayed diagnosis leading to more severe cases (adjusted odds ratio (OR) = 2.91, 95% CI: 2.17–3.89).^[74]^ While, others have, re-ported treatment outcomes similar to the pre-pandemic era.^[65,75]^ Among these, a study from Indonesia reported no increase in all-cause mortality despite an 11% decrease in treatment coverage for 2020 and 2021 (95% CI: 10–12).^[75]^

### 4.2. Unfavorable outcomes of TB infection

The 2024 global tuberculosis report estimates an excess of 670 thousand TB deaths from 2020 to 2023, as a result of the COVID-19 pandemic. The same report depicts a return to baseline mortality trends only in high-income countries.^[3]^ Similarly, data reported to the WHO from 49 high burden countries showed increased mortality in 2020 (RR = 1.08, 95% CI: 1.03–1.13), and increased treatment failure in both 2020 (1.14, 95% CI: 1.01–1.28) and 2021 (1.36, 95% CI: 1.03–1.78).^[76]^ Among high-burden countries Brazil and Indonesia are estimated to have suffered severe consequences, while China has experienced minimal impact.^[3]^

Several studies, mostly from low-burden countries, have found minimal negative impact for the COVID-19 pandemic on patient outcomes. Two cross-sectional studies conducted in Iran and Greece found no significant changes in TB mortality during the pandemic.^[77,78]^ Similarly, studies from South Korea, reported only minor delays in treatment, along with a transient increase in mortality in 2021.^[79,80]^ Studies from TB centers in Ethiopia and Lesotho, two high-burden countries located in Africa, also showed slight increase in mortality rate despite a meaningful drop in diagnosis and follow-up. This finding highlighted the value of ongoing outreach efforts.^[81,82]^

Meanwhile, other research has suggested a worsening of disease severity and out-comes during the pandemic. Despite a decrease in cases, a 24.3% increase in symptomatic presentations, and 25.9% in severe cases was observed in a pediatric center in Naples, Italy.^[83]^ A similar finding of increase in disseminated TB and delay to diagnosis was re-ported in a referral center from Barcelona, Spain.^[84]^ Treatment success in Peru, was also found to be significantly lower during the pandemic (OR = 0.81, 95% CI: 0.78–0.85).^[85]^

Multiple large-scale studies have also reported increased TB mortality during and after the pandemic. A report from Hong Kong found significant increases in treatment de-lay, ICU admission and mortality persistent up to December 2022^[86]^ A nationwide study from India found TB mortality in 2021 higher than that of 2020.^[87]^ Data from national reports in China showed a significant drop in TB mortality early on in the pandemic, followed by a rapid upslope – exceeding pre-pandemic rates in 2022.^[88]^ And, mortality in East Java, Indonesia was reported to have increased 36.9% during and 158.9% after the pandemic.^[56]^

These results can be attributed to an increase in treatment delays and disruptions, or, a true increase in TB incidence. Supporting the earlier proposition, a retrospective study from Kazakhstan found the adjusted risk of mortality during the pandemic to be 1.78 (95% CI: 1.41–2.26) times higher when compared to pre-pandemic results.^[42]^ And, a study from the Russian Federation reported a 4% increase in TB mortality, while expecting a steady decrease in the following years.^[89]^ 60% of the reported increase in mortality in Sao Paulo (5% increase 2020 and 12.7% in 2021) was also attributable to the newly diagnosed patients.^[90]^

Perhaps more concerning, a review of National Vital Statistics System data from the US showed age-standardized mortality increases of 10.2% and 9.2% in 2020 and 2021, especially among young and middle-aged patients.^[91]^ Child mortality in Indonesia was also found to have increased 116% in 2020.^[65]^ Meanwhile global estimates showed de-creased mortality among the elderly in 2020 and 2021.^[32]^ This pattern may have followed a disparity in observation of preventive public health measures.

### 4.3. TB transmission

A 30% to 70% drop in preventive treatment was observed in 24 countries studied in 2020^20]^ and a similar 50% to 80% decrease in preventive treatment was reported from Indonesia.^[35]^ These results indicate early and widespread disruptions in contact-tracing and sub-sequent provision of treatment to at-risk individuals. Although limited in time, these disruptions were severe. For example, Coutinho et al, reported a 93% drop in preventive treatment in Brazil up to July 2021 with subsequent recovery in early 2022.^[92]^ Following these disruptions, an increase in the proportion of patients reporting TB contacts was observed.^[93]^

Although close-contact transmission is a less common mode of TB transmission, it is of high importance among children and those living in overcrowded settings.^[4]^ Accordingly, disruptions in contact-tracing showed disproportionate impact on these vulnerable groups. Underdiagnosis was reported to be more severe among children (37.8%, 95% CI: 36.7–38.9 vs 21.8%, 95% CI: 21.4–22.1 for adults).^[86,94]^ And, in a review reporting a 5.9% to 14.3% increase in LTBI among household contacts of TB patients, 57% of children diagnosed with TB were found to have household contacts – a striking increase from the 5.3% reported in 2019.^[95]^ Similarly, a study from Catalonia, Spain found increased LTBI among children and adolescents, despite a 36% decrease in contact-tracing.^[96]^ These findings were replicated among populations deprived of liberty. With a 25% and 37.8% decreased contact-tracing in São Paulo during 2020 and 2021, there was a significant de-crease in prisoners diagnosed with TB (24.5% in 2021), while notifications increased by 68% among prison-workers.^[90]^

However, lockdowns did not impact all regions similarly. For example, TB cases in London were unchanged, unlike several other respiratory diseases – possibly due to low prevalence of LTBI and local transmission.^[97]^ Similarly, a study found stable con-tact-tracing in West Bengal, India through 2021, and a 34% decrease in secondary attack rate among contacts. These results were attributed to efficacy of public health measures instituted during the pandemic in decreasing TB transmission.^[98]^

Of interest, the pandemic may have decreased transmission of TB among healthcare workers (HCW) with extensive implementation of safety protocols and more common use of personal protective equipment (PPE). Kuwahara et al, reported a case where regular use of N95 masks during the pandemic prevented TB transmission to HCW.^[99]^ Later, a study from South Korea found the use of KF94 masks by HCW to be associated with a significant decrease in conversion rate to LTBI.^[100]^ A similar observation was made among HCW in Brazil.^[101]^

### 4.4. TB/COVID-19 co-infection

#### 4.4.1. Prevalence of co-infection

The prevalence of TB/COVID-19 co-infection has shown immense variation across regions and reports. The inconsistent method of TB diagnosis (LTBI, microbiological tests, molecular tests, etc.) has also caused divergence in reported results.^[102]^ Further complicating the matter, tests used in diagnosis of LTBI have been shown to yield false negative results in the setting of COVID-19.^[29,103]^ In a systematic review of relevant evidence, the prevalence of intermediate QuantiFERON-TB results among COVID-19 patients was found to be 26% (95% CI: 20–32), while this rate was suggested to be higher among those treated with corticosteroids or experiencing severe COVID-19.^[104]^

On a larger scale, a systematic review of more than 6000 COVID cases estimated the prevalence of TB co-infection to be 3.6% in Africa, 1.5% in Asia, and 1.1% in the Americas.^[105]^ And, an early pandemic study of South Korea’s National Health Insurance Service found no relationship between COVID-19 and TB diagnoses.^[106]^ Table 2 presents a summary of additional studies reporting the prevalence of TB-COVID-19 co-infection.^[107–115]^

**Table 2:** Prevalence of co-infection among tuberculosis patients.

Study region [Reference]	Test used for tuberculosis detection	Co-infection rate
Gül et al, Turkey^[107]^	Microbiological, or pathology	2.1%
Kishore et al, Brunei^[108]^	Not reported (active and latent tuberculosis)	5.7%
Yakupogullari et al, Turkey^[109]^	Microbiological, or molecular testing	15%
Mane et al, India, Pediatric^[110]^	Not reported	4.3%
Mangamba et al, Cameroon^[111]^	Microbiological, or molecular testing	24.3%
Morena et al, Spain^[112]^	Not reported	2.4%
Nabity et al, US^[113]^	Microbiological, molecular testing, or pathology	11.1%
Patel et al, India^[47]^	Not reported	5.1%
Sachdeva et al, India^[114]^	Microbiological, or radiology	5.2%
Negrao et al, Portugal^[115]^	Not reported	18%

#### 4.4.2. Outcomes of co-infection

Reports have been heterogeneous regarding the effect of co-infection on patient out-comes. An early cohort by the Global Tuberculosis Network presented 767 cases of co-infection from 34 countries, and reported an 11% mortality rate.^[116]^ Subsequently, several case-control studies from India, China and the US found no significant increase in ICU admission, need for mechanical ventilation, or mortality among patients with co-infection.^[117–119]^ Similarly, no increased mortality was reported among children co-infected with TB and COVID-19.^[110]^ At the same time, multiple studies have reported increased morbidity and mortality in co-infection.^[102,120–123]^ However, findings from several such studies are, at least, partially attributable to higher prevalence of comorbidities among TB patients.^[113,124–126]^ Table 3 depicts results from mentioned studies on outcomes of TB/COVID-19 con-infection.

**Table 3:** Methodology and findings of studies on outcome of Tuberculosis/COVID-19 co-infection.

Study design	Findings*; caveats	Reference; year
Case-control	No association with mortality (*P* value = .77)	Hazra et al, ^[117]^; 2023
	No association with mortality (*P* value = .63)	Hartandy et al, ^[118]^; 2023
	No association with mortality (*P* value = .16)	Lv et al, ^[119]^; 2023
Cohort	No association with mortality (*P* value = .50); pediatric population	Mane et al, ^[110]^; 2022
	Increased mortality among COVID-19 patients with “current TB” (RR = 1.47, 95% CI: 1.35–1.61); data from WHO global clinical platform of COVID-19 adjusted for age, sex, and comorbidities	Bastard et al, ^[121]^; 2024
	Increased mortality in COVID-19 patients with concurrent TB (HR = 2.62, 95% CI: 1.66–4.13); adjusted for age and sex	Easton et al, ^[122]^; 2023
	Increased COVID-19 mortality (OR = 3.02, 95% CI: 1.45–6.27); No adjustment for comorbidities	Lee et al, ^[123]^; 2024
	Increase in national COVID-19 CFR with increased incidence of TB (β = 3.15, 95% CI = 1.09–5.22)	Sanduzzi Zamparelli et al, ^[124]^; 2020
	Increased COVID-19 mortality with current (HR = 2.70, 95% CI: 1.81–4.04) and previous (HR = 1.51, 95% CI: 1.18–1.93) TB; adjusted for age, sex, and comorbidities	Luke et al, ^[125]^; 2020
	Increased mortality among patients with radiological evidence of TB sequelae (HR = 3.00, 95% CI: 1.34–6.73); No adjustment despite significantly higher comorbidities in patients with TB sequelae	Mariotti et al, ^[126]^; 2023
Meta-analysis	Increased COVID-19 severity (OR = 1.56, 95% CI: 1.13–2.16), and mortality (OR = 1.94, 95% CI: 1.28–2.93); No adjustment for comorbidities	Booysen et al, ^[102]^; 2021
	Increased COVID-19 mortality (RR = 2.10, 95% CI: 1.75–2.51); No adjustment for comorbidities	Shariq et al, ^[120]^; 2021

95% CI = 95% confidence interval, CFR = case fatality rate, HR = hazard ratio, OR = odds ratio, RR = relative risk, TB = tuberculosis, WHO = world health organization.

* *P* values reported for non-significant associations.

The role of previous TB, and associated pulmonary damage, in COVID-19 mortality remain ambiguous. We were able to find only one study reporting significant association between past TB and COVID-19 mortality.^[125]^ While two studies considering such an effect reported only associations between current TB and mortality.^[121,122]^

A systematic review conducted in February, 2022 found limited and low quality evidence on outcomes of co-infection.^[127]^ And a recent meta-analysis reported pooled mortality among patients to be 10.6% in patients with current TB (9.8% in-hospital mortality) and 5.7% among patients with past TB (21% in-hospital mortality), while relative risk analysis (based on data from 3 studies and 1285 patients) showed no increased in-hospital mortality among the co-infected (RR = 0.8, 95% CI: 0.18–3.68). Heterogeneity of patient characteristic in different groups, and the possible decrease in severity of newer COVID-19 strains was proposed to have affected these results.^[128]^

### 4.5. Post-COVID-19 tuberculosis

Few studies have quantified the possible risk of TB reactivation following COVID-19 infection. Due to low prevalence of TB in the general population, many small-scale studies reported the mere presence post-COVID-19 tuberculosis (PCT), and explored possible precipitating factors in narrative.^[25–27,109]^ Of such cases, many have had other risk factors for development of TB. For example, a study found more than two thirds of PCT cases had another major comorbidity, and 70% had used immunosuppressive drugs (corticosteroids or Tocilizumab).^[129]^ Alternatively, some reports have indicated lower prevalence of prior COVID-19 in TB patients compared to the general population.^[130]^

Among studies evaluating the relationship of a mere prior COVID-19 infection and TB diagnosis, most found no evidence suggesting such a link. A case-control study evaluating 296 pairs of TB and non-TB patients, found no significant evidence of PCT (OR = 1.45, 95% CI: 0.58–3.65).^[131]^ Similarly, two studies evaluating the association between COVID-19 seropositivity and TB diagnosis, found none.^[132,133]^ Furthermore, a model based on data from the East Java, Indonesia TB surveillance registry found no excess risk of TB among COVID-19 patients.^[56]^

Meanwhile, the bulk of evidence supporting a risk of PCT stems from severe COVID-19 cases. A case-control study found likely association between COVID-19 severity and possible subsequent PCT.^[134]^ Similarly, a large scale study on insurance claims da-ta in Thailand found significantly higher TB rates at 30 days among patients with a history of COVID-19 pneumonia (HR = 9.87, 95% CI: 5.64–17.30), and even symptomatic COVID-19 in longer follow-up (HR = 3.85, 95% CI: 2.42–6.13), but not in asymptomatic cases. Results were adjusted for HIV, diabetes, cancer, and chronic pulmonary obstructive disease, while authors estimated a diminishing role for the use of corticosteroids in their results.^[135]^ Furthermore, a longitudinal study showed 11-fold increase in TB after treat-ment of COVID-19 patients with corticosteroids – only at high doses exceeding the routine.^[136]^

## 5. Conclusions

TB notification trends during the pandemic were influenced by a complex interplay of social and ecological factors. There was a universal shock to TB reporting early in the pandemic. Subsequently, countries with low local transmission, stringent public health measures, and adaptive or reinforced TB control efforts seem to have experienced a true decrease in TB transmission and incidence. While, populations with low adherence to public health measures, high TB burden, and weak public health infrastructure suffered gravely with delays in diagnosis, and the ensuing increased TB morbidity and mortality. Further, the negative effects of increased close-contact exposure have disproportionately affected children, those living in poverty, or those deprived of liberty. These findings highlight a need for continued evaluation of the performance of impacted healthcare systems in TB control, and may warrant further specific outreach efforts for populations at-risk. Future research to evaluate the efficacy of such initiatives is crucial.

TB/COVID-19 co-infection may possibly impact patient outcomes negatively, but the presence of comorbidities among TB patients, and recent attenuations in COVID-19 sever-ty, decrease the significance of co-infection as a predictor of negative outcome. Finally, data suggesting an increased risk of TB due to COVID-19 infection is lacking, but for few results linking severe and symptomatic COVID-19 with TB reactivation.

## Author contributions

**Conceptualization:** Kiavash Semnani, Shirin Esmaeili.

**Data curation:** Kiavash Semnani, Shirin Esmaeili.

**Investigation:** Kiavash Semnani, Shirin Esmaeili.

**Methodology:** Kiavash Semnani.

**Writing – original draft:** Kiavash Semnani.

**Writing – review & editing:** Shirin Esmaeili.
